# Notes and News

**Published:** 1886-11-20

**Authors:** 


### Notes and News.

Collected and Communicated.

The Queen has sent to the Longmore Hospital for Incurables, Edinburgh, a donation of fifty pounds, and copies of her portrait for each of the rooms in the hospital.

Among "things not generally known," we may observe that Her Majesty the Queen was the first member of our Royal Family who received the benefit of Dr. Jenner's grand discovery. The little princess was vaccinated at Kensington Palace when she was three months old.

The Earl of Aberdeen has accepted the office of President of the Surgical Aid Society.

The Rev. Richard Hill, M.A., who for the past ten years has held the appointment of Chaplain to the South Western Fever Hospital at Stockwell, has been awarded by the Metropolitan Asylums Board a grant of £150 as compensation for the abolition of his office.

Mr. Robert Dyers Lysons, M.B., has been re-appointed for a further period of five years a member of the General Council of Medical Education and Registration for Ireland.

Bukovac, the distinguished painter, whose "White Slave" has recently attracted so much attention from the wonderful knowledge it displays of human anatomy, began life as a medical student.

The Brackley Cottage Hospital, which has been undergoing alterations and repairs, has just been re-opened. Two new wards, each capable on emergency of accommodating four patients, have been added, as well as a board-room, which is also intended to be used as a sitting-room for convalescent patients.

A new Eye Hospital, the want of which has long been felt in the South Wales district, is to be established at Cardiff. Patients will be treated gratis; two days a week, however, being set apart for those able to pay a small fee.

The Hospital Saturday Fund Collection closes on the 30th inst., by which date all outstanding amounts should be sent to the Central Office, 41, Fleet Street. Thus far, about 24,000 receipts have been issued.

A local District committee has just been formed with the object of raising funds towards the cost of the new buildings of the Great Northern Central Hospital in the Holloway Road. Officers were appointed, and the necessary arrangements made at a meeting recently held at Highgate, under the chairmanship of Mr. Francis Reckitt.

Every winter, at the Brompton Hospital for Consumption, musical entertainments are organised by the friends of the Institution, and afford much pleasure to those of the patients who are well enough to be present. The third concert of the present season was given on Tuesday.

Mr. Snarry, of Norton, Yorkshire, the distinguished veterinary surgeon, has performed another of his novel operations on cattle. This time he has amputated the hind-leg of a valuable shorthorn cow which had met with an accident. He has supplied the animal with an artificial limb, which, it is said, it is able to use with great ease. A few months back, it will be remembered, Mr. Snarry performed a similar operation, also with success.

On Monday next, the 22nd inst., a Scotch concert, in aid of the funds of the Scottish Hospital, will be given in the Surrey Masonic Hall, Camberwell New Road. Some youthful pipers from the Royal Caledonian Asylum will be amongst the performers.

A new hospital, called the "Mogg Memorial Hospital," and situated at Winterfield, near Paulton, was opened on the 9th inst. by the Bishop of Bath and Wells. It is intended as a memorial of the late Mr. John George Mogg of Farrington Gurney, and is built on a site given by Major Molyneux Carter from designs by Mr. W. F. Unsworth of Westminster. The stone of which it is built was given to the committee by the Prince of Wales, and the building is designed in the Queen Anne style.

The old Cottage Hospital at Paulton which was opened in 1872, had become inadequate to the needs of the surrounding district, notwithstanding the subsequent addition of a new ward for men, built at the expense of the late Lord Hylton, and bearing his name. The new hospital also contains a Hylton ward, furnished by the present peer.

The report read at the recent annual meeting of the subscribers of the Llanelly Hospital, which was completed and opened free from debt in December last, stated that on an average fifteen patients had undergone treatment every week for the last ten months. A general committee was elected at the same meeting, on the motion of the chairman, Mr. R. Maclaren.

Among the poor a vast amount of ignorance prevails on subjects appertaining to hygiene and medicine. Many a poor mother thinks if she can obtain a little physic at the dispensary, it is all that her suffering child requires. The dressing of a wound, the ventilation of a sick room, the proper diet for the patient, are all matters of vital importance, and herein lies the sphere of usefulness of the district nurse.

At a recent meeting of the Metropolitan Asylums Board, a statement was made by one of the managers that the present stock of champagne at the Eastern Hospital was valued at £100. As this statement seemed likely to be widely circulated, and to be too generally credited, we made it our business to ascertain the actual facts, and are now in a position to state that the stock of champagne, so far from representing £100 in value, may be estimated at barely one-fourth of that amount.

At the end of last month the Metropolitan Asylums Board appointed a Special Committee to consider and report upon the question of retaining the services of such of their officers as may not be required during non-epidemic periods.

The appointment of this Committee—which was not determined upon one moment too soon—was, we believe, in the first instance suggested by a consideration of the fact that at the present time no less than three of the six fever hospitals under the managers' control are, fortunately for the metropolis, not required for the treatment of fever patients, and are consequently closed; whilst the nursing staff on board the small-pox hospital ships at Long Reach—where there are at present only some three or four patients under treatment—has been reduced to a minimum.

This actual disuse of three out of the six fever hospitals, together with the great reduction effected in the staff of the floating Small-pox Hospital, has necessitated the turning adrift of many excellent nurses who have served the Board faithfully and well for periods ranging from five to fifteen years, and who have either been compelled to accept very inferior positions at other hospitals of the Board, or to seek a livelihood elsewhere.

The injustice of such a step is by no means confined to the nurses themselves, as might at first sight appear. The patients, too, must suffer; inasmuch as on the recrudescence of fever or small-pox the managers will be again compelled, as they have on every previous occasion, to engage nurses who are entirely ignorant of the disease which they are called upon to treat; whilst the services of those who have borne the heat and burden of previous epidemics, and who are thoroughly conversant with fever or small-pox, as the case may be, will not be procurable.

The West London Hospital at Hammersmith, which has been closed for several weeks past to allow of certain sanitary improvements and general repairs being carried out, will be reopened on the first of December for the admission of in- and out-patients. The complete closing of the hospital for so long a period has been most severely felt. Cleaning at most hospitals is, as a rule, done by degrees, only a certain number of wards being emptied at a time. It seems strange that a similar course was not adopted at this institution, especially when it is remembered that this is the only hospital within easy reach of Fulham, Hammersmith, and the adjacent populous suburbs.

There was a very sad case at the London Hospital the other day. A little boy, named William Hammond, was engaged last Friday at work in a coal yard at Bow Road Station of the Great Western Railway, when, by some means, he got caught between the buffers of two trucks. He was at once taken to the hospital, but his injuries soon terminated his sufferings. He was the eldest son of a very poor family (consisting of twelve children), living at Ernest Street, Stepney, and, although only thirteen himself, he assisted in their support.

The following vacancies are announced this week, the amount of the salary being placed in each case after the name of the institution :—

**Honorary Medical Officers.**

St. Marylebone General Dispensary. One vacancy.
Great Northern Central Hospital. Chloroformist.
North London Hospital for Consumption. Two Physicians.

**Resident Medical Officers.**

Dundee Royal Asylum. Qualified Assistant.—£80 with board.
Borough Hospital, Birkenhead. Doubly qualified.—£100 with board.
Rotherham Hospital. Assistant.—Board; no salary.
Moulsford Lunatic Asylum. Medical Superintendent.—£500, and house.
Salop Infirmary. Doubly qualified.—£100, with board.
St. Saviour's Union, Surrey. Doubly qualified.—£100.
Bootle Borough Hospital, Liverpool. Doubly qualified.—£80, with board.
Sussex County Lunatic Asylum. Junior Assistant Medical Officer.—£100, with board.
Morpeth Dispensary. Doubly qualified.—£120, and house.
Cheltenham Dispensary. Resident Surgeon.—£180, and house.

**Non-Resident Medical Officers.**

St. Saviour's Union, Surrey. Doubly qualified.—£130.

**Matrons.**

East Suffolk and Ipswich Hospital.—£60, with board.
Royal Hospital for Children and Women, S.E.—£40, with board.
Belfast Royal Hospital.—£65, with board.

**Trained Nurses.**

Cambridge Nurses' Home. Two.
Southport Infirmary.—£20, with uniform.
West Derby Union.—£20, apartments, uniform, and rations.
Establishment for Invalid Gentlewomen, Harley Street. Night.—£25.
Essex and Colchester Hospital. Male accident.—£25.
Great Northern Central Hospital. Head.—£20, with uniform.
Kent and Canterbury Hospital. Head.—£25, with board and uniform.
Kent and Canterbury Hospital. Surgical, Night, certificated. —£22, with board and uniform.
Hertford General Infirmary.—£16 to £20.

**Probationers.**

Establishment for Invalid Gentlewomen. One.
Dewsbury Infirmary. One.—£14, and uniform.

The first annual report of the Kensington Branch of the Metropolitan Association for nursing the sick poor at their own homes may, on the whole, be regarded with satisfaction. No profession has had its status so much raised during the past quarter of a century as that of the art of nursing, and it is now more than ever felt that, in the noble cause of humanity, the poor as well as the rich should have the inestimable services of the trained nurse. Wealthy as "Kingly Kensington" undoubtedly is, it nevertheless contains a large population of the poor, especially in the northern portion of the parish. The Central Association in Bloomsbury Square accordingly established a branch in Kensington, which was first housed in a house in the Earl's Court Road; then in Hornton Street, and finally in Gordon Place.

During its first year of operation, the Kensington District Nursing Association sent nurses to attend 168 poor patients (of whom 54 were dispensary cases) and no less than 3,204 visits were paid. The Metropolitan Association for Nursing the Sick Poor has now been at work about eleven years, and has homes at Holloway, Hampstead, Paddington, Wandsworth, Walworth, and Portsmouth. There is unquestionably a wide field of labour for district nurses. It is not every poor person who can be induced to go into the hospital, while others, again, though requiring skilled nursing, are not suitable cases for admission.

The article entitled "Dolly in the Hospital" which we published last week has excited a good deal of interest. Mr. Henry Labouchere, M.P., informs us that someone suggested the Toy Fund to him, and he took it up because he thought it a good one. We quite appreciate Mr. Labouchere's generosity, but we can never be too grateful to *Truth* for having made the Toy Fund such a splendid success.

---

The Santa Claus Society is, we are informed, independent of all other organisations, and is managed, with the assistance of a committee of ladies, by Miss J. F. Charles, of Hillside, Southwood Lane, Highgate, N., in whose family it originated. Miss Charles will be pleased to hear from any ladies who will assist by a present of toys for distribution among the children at our hospitals during the coming Christmas. Gifts of money would also be gratefully received and acknowledged.

---

The marvellous growth in the work and usefulness of cottage hospitals is made apparent by the facts stated in a special appeal put forth by the authorities of the Bromley Cottage Hospital. During the first few years of its existence the Bromley Cottage Hospital was located in two small cottages. In 1875 these were pulled down, and a hospital was erected on the site, which was granted by Mr. Alexander on a ninety-nine years' lease at a pepper-corn rental. The average number of cases treated for the first three years was thirty-two, but in the last three years they have been nearly one hundred, and many cases were refused admission for want of room. A new wing has just been added, from plans prepared by Mr. Ernest G. R. Peto, of Maddox Street, W., to whom we are indebted for a plan of the hospital, through the courtesy of Dr. H. J. Ilott, of Bromley. The recent additions have enabled a better classification of the sexes to be made, and the hospital now contains fifteen beds. The new buildings have cost about £2,000, and subscriptions are really needed and will be gratefully acknowledged by Dr. Ilott. We are glad to see the medical officers of the Bromley Hospital feel that the good results obtained have been in large measure due to the watchful supervision exercised by Miss Birmingham over the management and ventilation of the wards.

---

Mr. Newton H. Nixon, the secretary of University College Hospital, was quick to perceive the importance of securing the support of the working-classes. So long ago as January 1878 he successfully launched a People's Contribution Fund at a public meeting over which Sir Thomas Chambers, Q.C., M.P., presided. The movement had a two-fold object : (1) to increase the income of University College Hospital, and (2) to enable the working-classes to secure the hospital treatment they might need from time to time without loss of wages or time, and free from delay and difficulty. No wonder that the people's fund has prospered and increased. In the first twelve months over three hundred pounds was realised, and during 1885-86 more than six hundred pounds (£614) has been handed over by the fund to the hospital. This is gratifying proof of the literal accuracy of what H.R.H. the Prince of Wales said last May of the People's Fund: "it is doing excellent service, and its success affords much gratifying evidence of the sympathy of the working-classes in the welfare of University College Hospital." Mr. Nixon and all concerned are to be congratulated upon this genuine success, and we commend the movement to the notice of the managers of all the metropolitan hospitals.

---

The Honourable Maria Eden writes from Boyn Hill House, Maidenhead, to say that her Convalescent Home at Maidenhead is not full at this time of the year, and that she would be glad to have this known, in case it might be of use to persons able to pay for themselves, and who require change of air. The payments are seven shillings weekly, except to members of the G. F. S., who pay six shillings, exclusive of travelling and washing expenses. A train leaves Paddington daily at 2.30 p.m. for Maidenhead Station. The railway fare, third-class, is 2*s.* 0½*d.*, and the cab from the station to the Home, which is close to All Saints' Church, Boyn Hill, costs eighteenpence. Infectious cases and fits are inadmissible, as are those who require medical treatment and nursing.

---

The late Canon Morse inaugurated the Hospital Saturday and Sunday collections at Nottingham, the former eighteen and the latter thirteen years ago. Canon Morse was chairman of the weekly Board of the Nottingham General Hospital for eight years, and at the annual meeting of the Governors it was stated by Earl Manvers, who presided, that these collections had brought to the hospital no less than £32,825, a sum which represented the relief of seven thousand in-patients, who otherwise must have been excluded from the benefits of the institution. No wonder Canon Morse will be missed, or that Earl Manvers should declare that he had left his mark on the town as no vicar of St. Mark's had ever done before.

---

The management of the Nottingham General Hospital seems to be economically excellent. In 1881-82, 1,215 out-patients and 7,224 in-patients were treated at a cost of £7,046. In 1885-86 the expenditure had fallen to £6,439, although, compared with the former year, four hundred more out-patients and fifteen hundred additional in-patients received treatment. We are no believers in the mistaken economy of some hospital reformers, who are always urging the practicability of curing a greater number of patients at a greatly reduced cost every year. Such a system, if pushed to its logical conclusion, must end in the treatment of the entire population without any expenditure of money at all. True economy consists, of course, in finding the irreducible minimum of expenditure, having a due regard to efficiency and treatment, and then in working a hospital on this basis. The Nottingham General Hospital is deservedly popular with the patients, as the report testifies, and the reduction in expenditure there, is, for this reason, matter for sincere congratulation and general imitation. No mention is made in the report of Miss Rimington, the lady superintendent, or the other officers, all of whom deserve credit for the general efficiency of the management of the institution.

